# Developing a CRISPR/FrCas9 system for core promoter editing in rice

**DOI:** 10.1007/s42994-024-00157-5

**Published:** 2024-04-22

**Authors:** Hui Wang, Jian Ding, Jingyan Zhu, Xiaoshuang Liu, Rongfang Xu, Ruiying Qin, Dongfang Gu, Min Li, Pengcheng Wei, Juan Li

**Affiliations:** 1 College of Agronomy, Anhui Agricultural University, Hefei, 230036 China; 2 Key Laboratory of Rice Genetic Breeding of Anhui Province, Rice Research Institute, Anhui Academy of Agricultural Sciences, Hefei, 230031 China; 3 Research Centre for Biological Breeding Technology, Advance Academy, Anhui Agricultural University, Hefei, 230036 China

**Keywords:** Genome editing, FrCas9, CRISPR, Core promoter, Base editing

## Abstract

Small mutations in the core promoter region of a gene may result in substantial changes in expression strengths. However, targeting TA-rich sequences of core promoters may pose a challenge for Cas9 variants such as SpCas9 and other G-rich PAM-compatible Cas9s. In this study, we engineered a unique FrCas9 system derived from *Faecalibaculum rodentium* for plant genome editing. Our findings indicate that this system is efficient in rice when the TATA sequence is used as a PAM. In addition, FrCas9 demonstrated activity against all 16 possible NNTA PAMs, achieving an efficiency of up to 35.3% in calli and generating homozygous or biallelic mutations in 31.3% of the T_0_ transgenic plants. A proof-of-concept experiment to examine editing of the rice *WX* core promoter confirmed that FrCas9-induced mutations could modify gene expression and amylose content. Multiplex mutations and deletions were produced by bidirectional editing, mediated by FrCas9, using a single palindromic TATA sequence as a PAM. Moreover, we developed FrCas9-derived base editors capable of programmable conversion between A·T and G·C pairs in plants. This study highlights a versatile FrCas9 toolset for plant core promoter editing, offering great potential for the fine-tuning of gene expression and creating of new germplasms.

**Supplementary Information:**

The online version contains supplementary material available at 10.1007/s42994-024-00157-5.

Dear Editor,

In recent decades, the use of CRISPR-Cas9 genome tools has led to substantial advancements in crop research. These tools have been utilized to create programmable knockout mutations or single-nucleotide polymorphisms (SNPs) in the gene coding region, aiming to improve specific traits (Gürel et al. 2020). However, considering that agronomically important traits are mostly quantitative, there is growing interest in editing promoters to alter the strength of gene expression (Shi et al. 2023). Eukaryotic promoters consist of a core promoter region and upstream cis-regulatory elements (CREs). Successful editing of CREs has created a series of desirable traits that were previously absent in natural resources (Liu et al. 2021; Rodríguez-Leal et al. 2017; Song et al. 2022; Zhou et al. 2023). Nevertheless, achieving gene expression alterations through the targeting of multiple CREs is often laborious. Alternatively, minimal mutations in the core promoter region, which are replete with short TA-rich sequences, can induce significant changes in the abundance of downstream gene transcripts. Unfortunately, due to the lack of an appropriate protospacer adjacent motif (PAM), G-rich PAM recognition Cas9 enzymes, such as *Streptococcus pyogenes* Cas9 (SpCas9), are rarely used for core promoter editing.

Various Cas12a proteins and members of the Cas12 family preferentially utilize T-rich PAMs (Gürel et al. 2020). Although CRISPR-Cas12 systems have been extensively applied for single- and multiple-gene editing, base editing, gene activation, transcriptional repression, epigenome editing, and promoter editing (Cheng et al. 2023; Liu et al. 2022; Ming et al. 2020; Zhang et al. 2021; Zhou et al. 2023), the scope may be restricted by the requirement of relatively longer PAMs. The Cas9 gene of the probiotic *Lactobacillus rhamnosus* (LrCas9) and the hybrid SpCas9–*Streptococcus macacae* Cas9 enzyme (iSpyMacCas9) have expanded the editing scope to include NGAAA and NAA PAMs in crops (Sretenovic et al. 2021; Zhong et al. 2023). The recent discovery of a type II-A Cas9 ortholog (FrCas9) from *Faecalibaculum rodentium* provides another viable solution, due to its compatibility with a TATA PAM (Cui et al. 2022), specifically the consensus Goldberg–Hogness (or TATA) box sequence of core promoters. However, the activity of FrCas9 and its editing pattern have not yet been established in plants. In the present study, we engineered FrCas9 tools for rice genome editing and demonstrated the potential for modifying plant core promoter regions.

To engineer a FrCas9 tool for plants, the coding sequence was optimized, according to the rice codon preference, and placed under the control of the maize ubiquitin (ZmUBI) promoter in a pHUC400 binary vector backbone (Fig. 1A). To achieve maximum efficiency, an artificial scaffold with 3’ crRNA truncation was chosen to construct a sgRNA with a 22 bp guide sequence (Fig. S1). The sgRNAs were expressed with the Pol II CmYLCV promoter, and the transcripts were processed with Gly-tRNA and an HDV ribozyme to ensure the accuracy of the sequence (Fig. 1A).

**Fig. 1:**
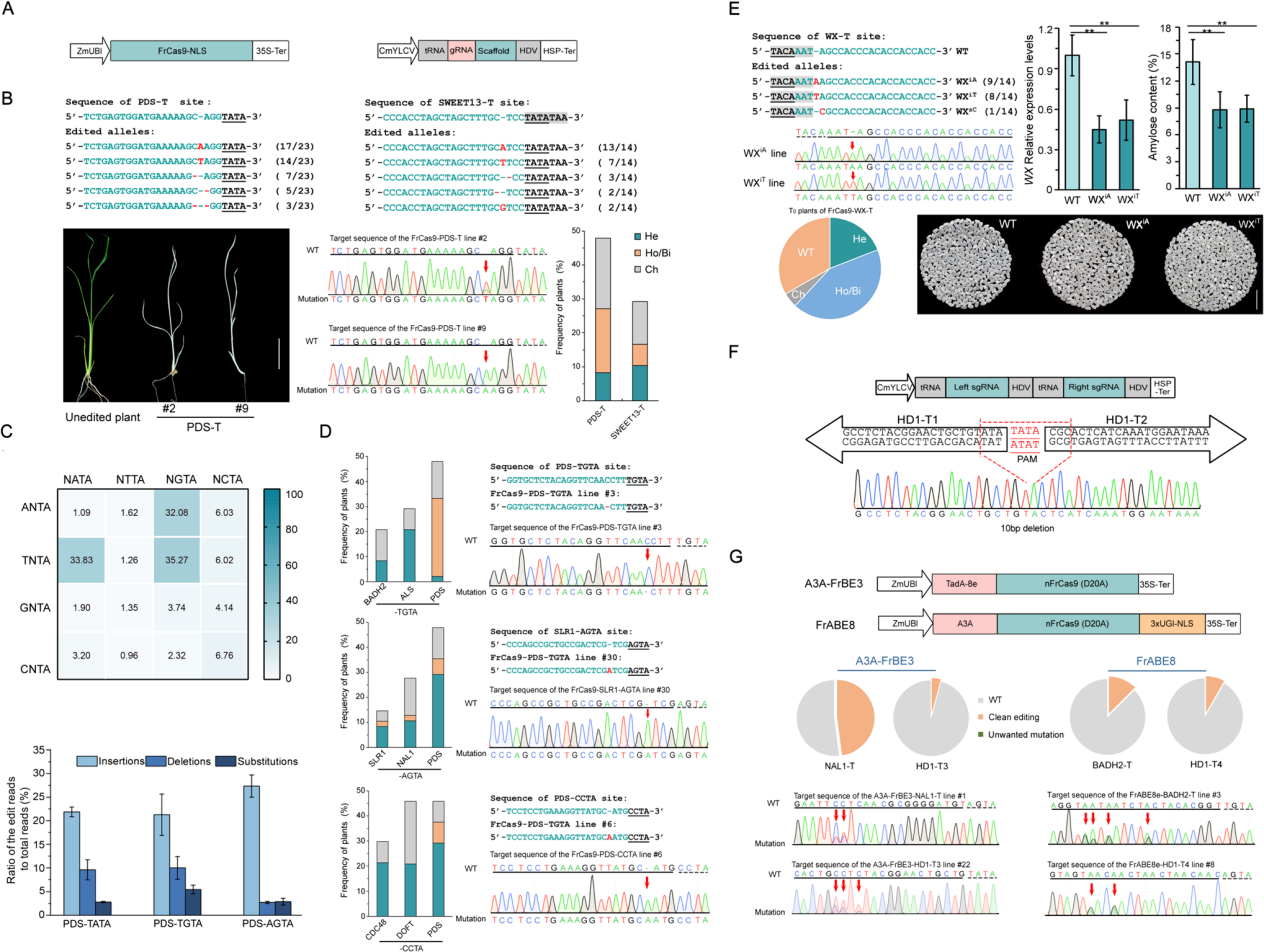
The CRISPR/FrCas9 system mediates genome editing in rice. **A** Schematic illustration of the FrCas9 and sgRNA expression cassettes used for plant editing. Left, the ZmUBI promoter and the CaMV *35S* terminator (35S-Ter) were used for FrCas9 expression. NLS, SV40 nuclear location signal. Right, a tRNA (Gly-tRNA) and an HDV ribozyme were built in the sgRNA cassette driven by the CmYLCV promoter. HSP-Ter, Arabidopsis heat shock protein 18.2 terminator. **B** FrCas9-induced targeted mutations of the TATA PAM in the *OsPDS* and rice *OsSWEET13* genes in T_0_ transgenic plants. Top, frequently occurring mutation alleles produced by the plant FrCas9 system. Red -, deleted nucleotides; red letters, insertions. The number of plants that carried the corresponding edit is noted on the right. The PAM sequences are underlined. Bottom, representative albino T_0_ transgenic lines produced by the FrCas9 system at the PDS-T site. Scale bar, 2 cm. Sanger sequencing chromatographs of representative albino lines at the PDS-T site; homozygous or biallelic mutations were obtained. WT, wild type. Red arrows indicate mutations. The occurrence frequencies of homozygous (Ho) or biallelic (Bi), heterozygous (He), and chimeric (Ch) mutants of PDS-T and SWEET13-T are shown. **C** FrCas9 editing of NNTA PAMs and outcome preferences in rice. Top, heatmaps of FrCas9-induced mutagenesis at all possible NNTA PAMs at the *OsPDS* locus. The mean efficiencies from three biological replicates are presented. Bottom, the preference of FrCas9-induced mutagenesis for insertions, deletions, and substitutions at the targets with TATA, TGTA, and AGTA PAMs. The efficiency was calculated by counting reads of mutation compared with the total clean reads. **D** Identification of targeted mutants in CRISPR-FrCas9 transgenic plants at the target sites with TGTA, AGTA, and CCTA PAMs. For each sgRNA, 48 independent T_0_ lines were randomly selected and genotyped. The ratio of the number of mutants to the total number of plants was considered the frequency. Representative sequence alignments and Sanger sequencing of the targeted mutants are shown on the right. **E** FrCas9-mediated core promoter editing of rice *WX* with the TATA box as the PAM. Left, FrCas9-edited alleles of the *WX* core promoter. The TATA box is shaded. The homozygous lines were confirmed by Sanger sequencing. The zygotypes of the targeted mutants were determined by Hi-TOM assays. Right, the *WX* expression and amylose content of grains in T-DNA-free homozygous T_1_ mutants are indicated. Three independent lines of each allele were used as biological replicates. The significance of the difference was determined by a two-tailed *t* test (**P < 0.01). The appearances of the grains of the *WX* core promoter mutants were compared with those of the WT. Scale bar, 1.5 cm. **F** Illustration of FrCas9-induced bidirectional editing. The designs of the left sgRNA (HD1-T1) and the right sgRNA (HD1-T2) for a single TATA PAM (red) in the rice *HD1* gene are indicated. The putative cleavage sites are labeled with red dashed lines. A representative sequencing chromatograph of the FrCas9-induced 10-bp deletion is shown. **G** Base editing induced by FrCas9-derived BEs in rice. Schematic representation of the expression cassettes of A3A-FrBE3 and FrABE8e. The frequencies of A3A-FrBE3- and FrABE8-induced base editing in plants are presented. All edited plants carried clean A-to-G or C-to-T conversions, while byproducts, such as indels, were not found in T_0_ plants. For each target, Sanger sequencing of a representative line is shown

To validate the core promoter editing capability of the FrCas9 system, the TATA box sequence was used as a PAM to design a sgRNA at the proximal promoter of a susceptibility gene *OsSWEET13* of Nipp (*Oryza sativa* L. cv *Nipponbare*) to *Xoo*. Moreover, to directly monitor editing activity, a sgRNA was designed to target the *PDS-T* site at the sixth exon of the *rice phytoene desaturase* (*OsPDS*) gene. These vectors were then introduced into Nipp cultivar plants, via Agrobacterium-mediated transformation. Out of the 48 PDS-T transgenic lines, six albino plants were obtained (Fig. 1B), suggesting that the FrCas9 system is active in rice. Subsequent sequencing confirmed that all the albino plants carried homozygous or biallelic mutations at the target site (Fig. 1B). Afterwards, the remaining T_0_ transgenic lines were analyzed through Hi-TOM sequencing. The data revealed that 47.9% of the total plants were edited by FrCas9 at the *PDS-T* target (Fig. 1B). Similarly, targeted mutations were observed in 29.2% of the transgenic lines at *SWEET13-T* (Fig. 1B). Homozygous or biallelic mutations were detected in 6.3% of the T_0_ plants at the promoter target, which enabled rapid evaluation of gene expression editing in early generations. At both targets, mutations at position 19 of the protospacer sequence (counting from the end distal to the PAM as position 1), which is the putative cutting site of FrCas9, were the most frequently observed edits (Fig. S1). Single-nucleotide insertions were the predominant type of mutation, occurring in an average of 93.3% of the edited lines (Fig. S2). Taken together, these preliminary results indicated that FrCas9 could induce targeted mutagenesis in the gene coding sequence and regulatory region of the plant genome.

In TATA-less promoters, short TA-rich sequences in the core promoter region frequently stretch functions to initiate transcription (Sugihara et al. 2010). To evaluate the potential of FrCas9 to edit TA-flanked sequences, 16 sgRNAs with all possible canonical 5’-NNTA-3’ PAMs were designed for the *PDS* gene locus. Amplicon next-generation sequencing (NGS) revealed that all 16 sites in stably transformed rice calli were edited with varying efficiencies, ranging from 1.1 to 35.3% (Fig. 1C). These findings demonstrated the high PAM compatibility of FrCas9, thus providing a wide range of opportunities to edit TA-rich core promoters in plants. In rice cells, FrCas9 exhibited a top efficiency of 35.3%, 33.8%, and 32.1% at the site with TGTA, TATA, and AGTA PAM, respectively (Fig. 1C), which is consistent with the presumed preference for *R* (*R* = *A*/*G*) at the second nucleotide of the NNTA PAM (Cui et al. 2022). A detailed outcome analysis of the editing efficiency at the three sites showed that, on average, 28.2% of the total reads harbored mutations at position 19 of the protospacer (Fig. S3). Insertions were the main types of edits, accounting for 21.3% to 24.7% of the total reads (Fig. 1C). Like in the case of the mutation type preference in transgenic plants described above, the majority of the insertions were 1 bp in length (Fig. S4), likely resulting from repair of the 1-nt 5' overhang of cleavage 3 bp upstream of the PAM. Given that a variety of small indels commonly occur during SpCas9 editing, these results imply that FrCas9 may have a distinct cutting pattern in plants. Interestingly, A or T insertion was preferred for more than 84.9% of the 1-bp insertions (Fig. S4), confirming a recently reported DNA Pol λ-mediated specific repair pattern of 1-nt overhangs in plants (Zhang et al. 2024). The editing profile suggested that FrCas9 may serve as a precise insertion tool for plant genome editing.

To validate the editing performances, FrCas9-induced mutagenesis was further examined in transgenic plants at three genomic sites with each of the favored PAMs TGTA and AGTA, as well as at sites with the less favorable CCTA PAM. The desired mutants were successfully obtained at all the sites, with average frequencies of 32.6%, 30.1%, and 20.2% at the TGTA, AGTA, and CCTA PAM sites, respectively (Fig. 1D). Remarkably, as many as 31.3% of the plants (at the PDS-TGTA site) carried homozygous or biallelic mutations. In addition, the potential sgRNA-dependent off-target effects of the plant FrCas9 system were also evaluated for four highly effective sgRNAs of PDS-T, PDS-TGTA, PDS-AGTA, and PDS-CCTA. Hi-TOM assays revealed no mutations at potential off-target sites with up to 5 nt mismatches in the T_0_ rice lines (Table S2), which is consistent with the high specificity of FrCas9 in HEK293T cells (Cui et al. 2022). Taken together, these results indicate that the plant CRISPR-FrCas9 system holds promise as an alternative tool for editing targets inaccessible to SpCas9.

To test the core promoter editing ability of FrCas9, a sgRNA was designed with the TGTA PAM at the predicted atypical TATA box (TACAAAT) sequence in the promoter of the rice *Waxy* (*WX*) gene. Of the 21 regenerated transgenic lines, 14 exhibited mutations in the core promoter region (Fig. 1E). The homozygous mutants resulting from a single A or T insertion, which were the two main outcomes of the editing process, were selected and designated WX^iA^ or WX^iT^, respectively. The mutations were faithfully transmitted to offspring, and T-DNA-free T_1_ plants were obtained for analysis. The transcript abundance of *WX* in the WX^iA^ and WX^iT^ lines was 45.4% and 52.1% lower than that in the wild type, respectively (Fig. 1E). This confirmed that the small mutation in the TATA box could lead to a significant difference in gene expression. As expected, the mutants showed average 37.6% reduction in amylose content (AC) compared to that in a *WX*^*b*^ Nipponbare variety. Therefore, FrCas9-mediated core promoter editing is a reliable strategy for creating novel germplasms via fine-tuning gene expression.

Due to the palindromic nature of the TATA sequence, bidirectional editing by FrCas9 was performed by coexpressing two sgRNAs for a single PAM (Fig. 1F, Table S2). Screening of the regenerated lines revealed successful editing in 18 out of 24 plants. Among them, ten plants exhibited simultaneous edits on both sides of the PAM, indicating the expanded scope of FrCas9. In addition, three plants carried deletions across the PAM, one of which had a precise 10-bp deletion between the putative cleavage sites targeted by the two sgRNAs (Fig. 1F).

Base editors (BEs), commonly derived from SpCas9 or its variants, have been utilized to enhance agronomic traits in various plants. To offer alternative BEs for TA-rich regions, fusions were created by combining the FrCas9 D20A nickase with the highly active cytidine deaminase APOBEC3a and the evolved adenine deaminase TadA-8e (Fig. 1G). Two targets were assessed for each of the resulting A3A-FrBE3 and FrABE8e editor in the T_0_ transgenic lines (Table S3). C-to-T conversions induced by A3A-FrBE3 were obtained at the NAL1-T site in up to 47.9% of the plants, while editing occurred at the HDT1-T site with a frequency of 4.2% (Fig. 1G). For FrABE8e, single and multiple A-to-G edits were produced across a wide window from position 5 to position 13 in 8.3% and 12.5% of the lines (Fig. 1G). While the efficiency may vary among sites, no byproducts were detected in the assays, demonstrating the high accuracy of the FrCas9-BE system.

The core promoter plays a direct role in controlling the abundance of transcription independent of spatiotemporal regulation (Grishkevich et al. 2011). Minor modifications in the core promoter can result in highly variable promoter strengths, making it an ideal target for modifying gene expression. In this study, a plant genome-editing system was engineered from the high-fidelity FrCas9. This plant FrCas9 toolset enables the generation of small indels, fragment deletions, and base conversions in rice genomic targets with NNTA sequences, such as the TATA box, as PAMs, which is widespread in core promoter regions. Given that the TATA box is typically situated ~ 30 bp upstream of the transcription start site, editing with this palindromic PAM in either direction could introduce mutations in the core promoter. A proof-of-concept experiment verified that modifying the TATA box in the core promoter via FrCas9 editing resulted in core promoter mutants of *WX*, which influenced rice grain quality by manipulating gene expression levels (Fig. 1F). Considering that FrCas9 exhibits efficiency comparable to that of SpCas9 in mammalian cells (Cui et al. 2022), it is reasonable to expect that an optimized FrCas9 could serve as a promising platform for promoter editing to create favorable crop germplasms.

A new set of FrCas9 tools was developed to expand targeted mutagenesis and base editing to TA-rich sequences of the plant genome. These systems offer a powerful strategy for adjusting agronomic trait performance through core promoter editing with the TATA box as a PAM in crops.

## Materials and methods

### Vector construction

The coding sequence of FrCas9 was codon optimized for rice expression (Supplemental sequence) and synthesized (GenScript, Nanjing, China). It was inserted into a pHUC backbone via *Pst*I/*Sac*I double digestion. Moreover, the single guide RNA (sgRNA) scaffold was synthesized and recombined into a CmYLCV promoter-driven cassette (Li et al. 2022) using Gibson assembly with a Hi-Fi cloning mixture (NEB, Ipswich, USA). The sgRNA expression component was subsequently integrated into the pHUC-FrCas9 vector via digestion with *Hin*dIII. A spectinomycin resistance gene (SpR) was preloaded in the sgRNA expression cassette as a negative selection marker.

To construct base editors (BEs), the D20A mutation was introduced into FrCas9 using a Fast Mutagenesis System (TransGen, Beijing, China). The sequences of APOBEC3A (A3A), three copies of uracil DNA glycosylase inhibitor (3 × UGI), and TadA-8e were cloned from SpG-BEs (Li et al. 2021). The BEs of FrCas9 were assembled using the Gibson method following previously reported methods (Li et al. 2021).

The binary vectors were linearized using *Bsa*I to release the SpR marker. Forward and reverse oligos of 22-nt guide sequences and 5′ overhangs were synthesized (Sangon, Shanghai, China) and annealed for single sgRNA ligation. A double sgRNA expression cassette was assembled using Golden Gate cloning. The sequences of the vectors were confirmed by Sanger sequencing.

### Rice transformation and plant growth

The vectors were subsequently introduced into *Agrobacterium* EHA105 using the freeze–thaw method. The clones were confirmed through colony PCR and PCR sequencing of the sgRNA cassette. For the analysis of FrCas9 activity at the 16 NNTA PAMs, at least three positive clones were selected as biological replicates. Rice transformation was performed by inducing embryonic calli from mature seeds of the rice variety Nipponbare for 2–3 weeks and infecting them with an Agrobacterium suspension. A minimum of 300 calli were recovered and selected under 50 mg/L hygromycin. After 4 weeks of selection, three to four resistant calli from each resistance event were transformed to regenerate plants. For each independent resistance event, only one shoot was rooted to produce an independent T_0_ line for the assays.

The edited T_0_ plants were grown in a greenhouse at 26–30 °C with a 16/8 h light/dark photoperiod. The seeds of the WX-edited T_0_ plants were germinated on soil in the greenhouse on April 15th, 2023. The T-DNA-free T_1_ lines were subsequently transferred to an experimental field in Hefei on May 22nd. Mature seeds were collected on September 5th for further analysis.

### Sampling and genotyping

To assess the editing efficiency of FrCas9 at the 16 NNTA PAMs, calli were transformed and selected for 2–3 weeks. The newly emerged calli from at least 200 independent events for each replicate were collected as one sample. Genomic DNA was isolated using a Plant Genomic DNA Isolation Kit (Tiangen Biotech, Beijing, China). The target amplicons were examined through the PE150 system of the Illumina HiSeq X Ten platform (Sangon, Shanghai, China). At least 0.5 Gb of data were obtained and analyzed using CRISPResso2.

To genotype a plant, leaves from three different tillers of one line were collected for DNA extraction using the cetyltrimethylammonium bromide (CTAB) method. The mutations were examined via NGS-based high-throughput tracking of mutation (Hi-TOM) assays with a 10% threshold (Liu et al. 2019). For the albino PDS-T lines and other representative edited plants, Sanger sequencing was performed. Some chromatographic overlaps were resolved through sequencing TA clones of the target amplicon. All primers used in this study are listed in Supplemental Table S4.

### Gene expression analysis

The expression of *WX* was analyzed in the grains of the wild-type (WT) and T-DNA-free homozygous T_1_ mutant plants after 14 days of filling. Three independent lines of each allele were used as biological replicates. Total RNA was extracted from ~ 0.2 g of sample using TRIzol reagent (Invitrogen, Carlsbad, USA). Reverse transcription of cDNA was performed using HiScript III All-in-one RT SuperMix Perfect (Vazyme, Nanjing, China). Quantitative PCR was conducted using the qTOWER 2.2 system (Analytic Jena, Jena, Germany) and M5 HiPer Realtime PCR Super Mix (Mei5bio, Beijing, China). Relative gene expression levels were calculated using the 2^-∆∆CT^ algorithm, with the rice *ACTIN2* gene serving as the internal control.

### Determination of amylose content

The mature seeds were ground into flour using steel beads. Approximately, 0.1 g of flour from a sample was dissolved in a 0.09% sodium hydroxide solution. The amylose content was then determined through flow injection analysis with an iodine reagent using an automatic analyzer (HACH, Loveland, USA). The absorbances of the resulting colors were recorded at 720 nm for content calculation.

### Supplementary Information

Below is the link to the electronic supplementary material.

Supplementary file1 (DOCX 288 KB)

## Data Availability

The deep sequencing data obtained during the current study are available in the NCBI database under the SRA accession numbers PRJNA1026048 and PRJNA1025872. All the data generated during this study are included in the article and its supplementary information files.
