# Supplementary material for: Developing a CRISPR/FrCas9 system for core promoter editing in rice
Source: aBIOTECH. 2024 Apr 22;5(2):189–95. doi: 10.1007/s42994-024-00157-5 (PMC11224051; doi:10.1007/s42994-024-00157-5)
Supplement: Supplementary file 1 — Supplementary file1 (DOCX 288 KB) [file 42994_2024_157_MOESM1_ESM.docx]

**Supplemental materials**

1. **Supplemental Figure S1.** Schematic representation of the sgRNA structure for FrCas9.
2. **Supplemental Figure S2.** The outcome preference of FrCas9-mediated mutagenesis on TATA PAM targets in transgenic plants.
3. **Supplemental Figure S3.** Position preference of FrCas9-mediated mutations in rice.
4. **Supplemental Figure S4.** Outcome preference of FrCas9-mediated editing in rice calli.
5. **Supplemental Table S1.** Determining potential off-target mutations mediated by FrCas9 in transgenic plants.
6. **Supplemental Table S2.** FrCas9-mediated bidirectional editing with a single PAM in transgenic rice plants.
7. **Supplemental Table S3.** Editing conducted by FrCas9-based BEs in T_0_ plants.
8. **Supplemental Table S4.** Oligos and primers used in this study.
9. **Supplemental sequences.**

**
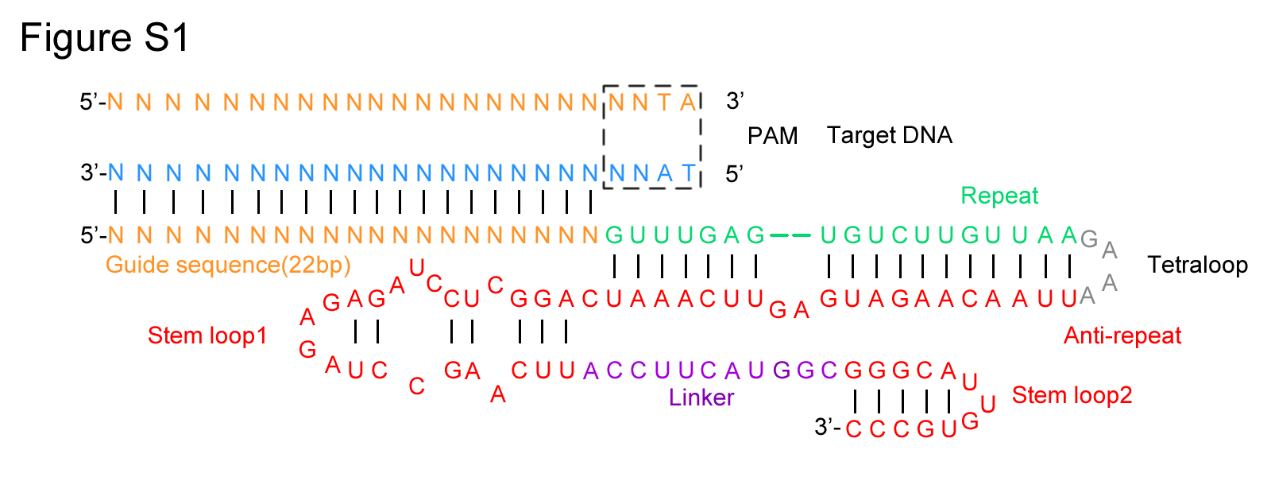
**

**Supplemental Figure S1.** Schematic representation of the sgRNA structure for FrCas9.

The PAM is indicated by the dashed box in the target DNA. Orange, guide sequence; red, stem loop and anti-repeat sequence; green, repeat sequence; purple, linker. The sgRNA used in the study was optimized from the tracrRNA-crRNA complex of FrCas9.

**
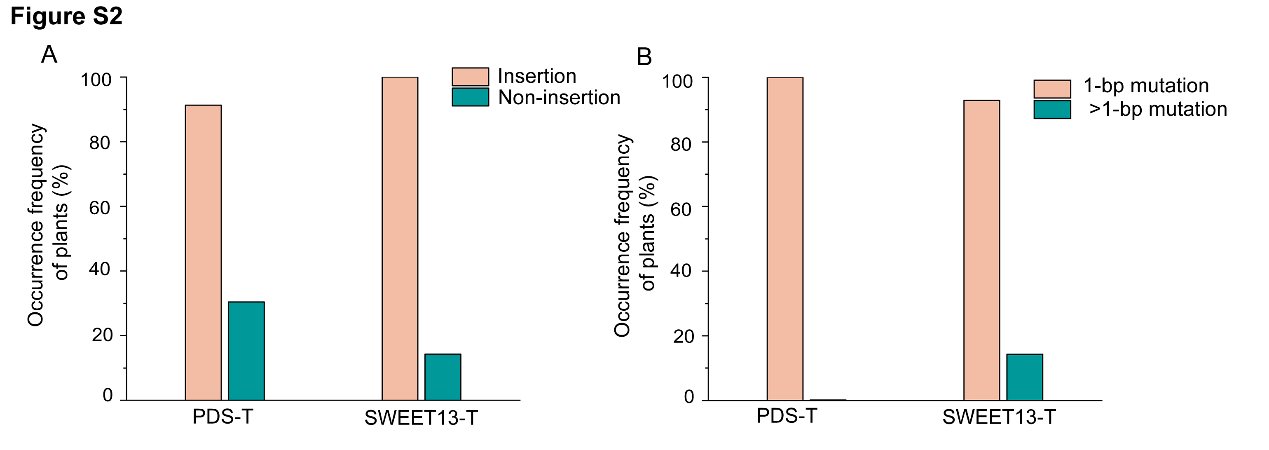
**

**Supplemental Figure S2.** The outcome preference of FrCas9-mediated mutagenesis on TATA PAM targets in transgenic plants.

The occurrence frequencies of the edit types were calculated from the ratio of plants that carried insertions/no insertions (A) or 1-bp/non-1-bp mutations (B) to total mutants. Notably, biallelic and chimeric mutants could simultaneously carry insertions and no insertions or 1-bp and non-1-bp mutations in one line.

**
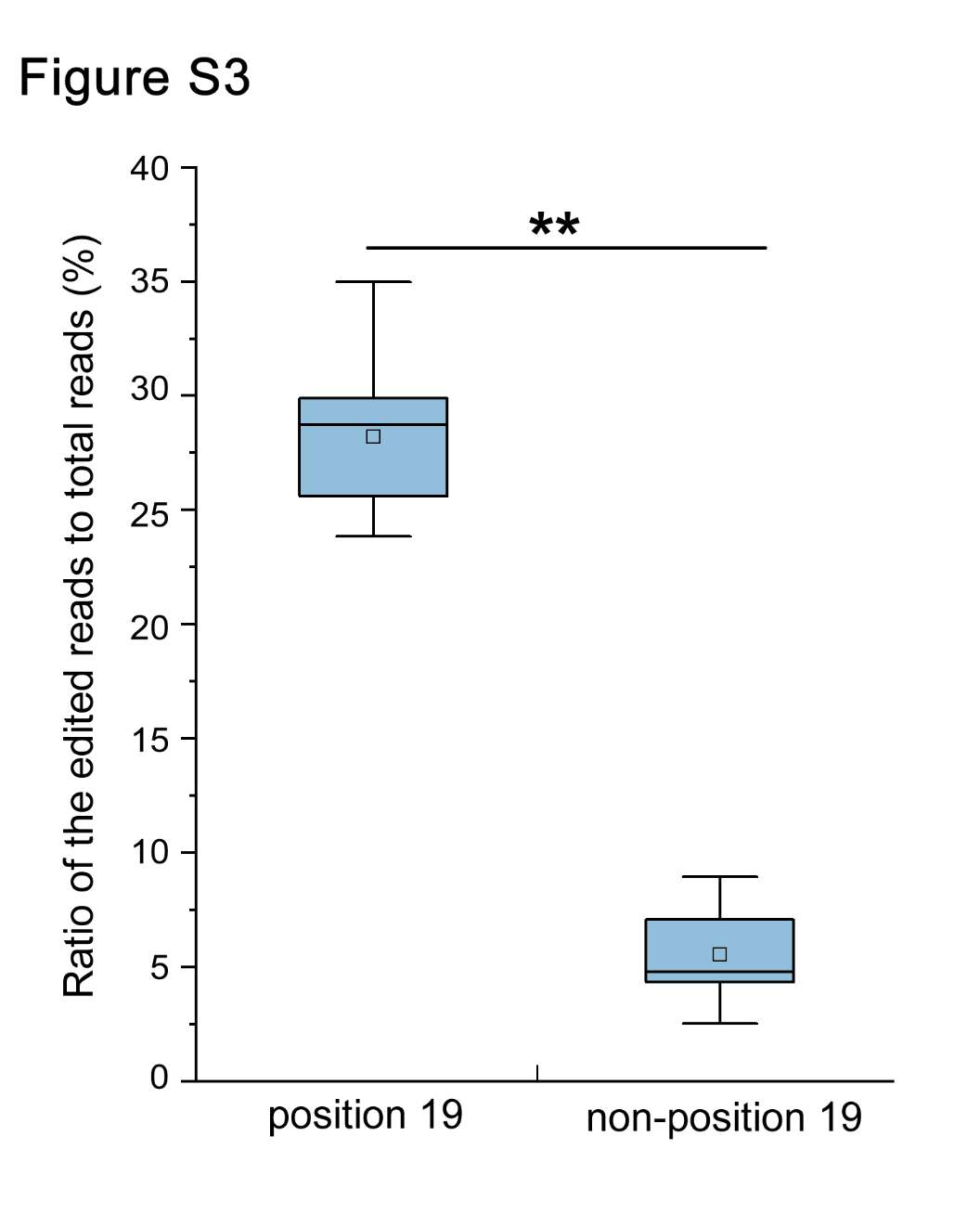
**

**Supplemental Figure S3.** Position preference of FrCas9-induced mutations in rice.

The mutations at the PDS-TGTA, -AGTA, and -CCTA sites were analyzed. The ratios were calculated from the reads mutated at position 19 or at other positions of the protospacer sequence to the total reads. The line represents the median value, and the square represents the mean value. Significance was determined by one-way ANOVA (**, *P*<0.01).

**
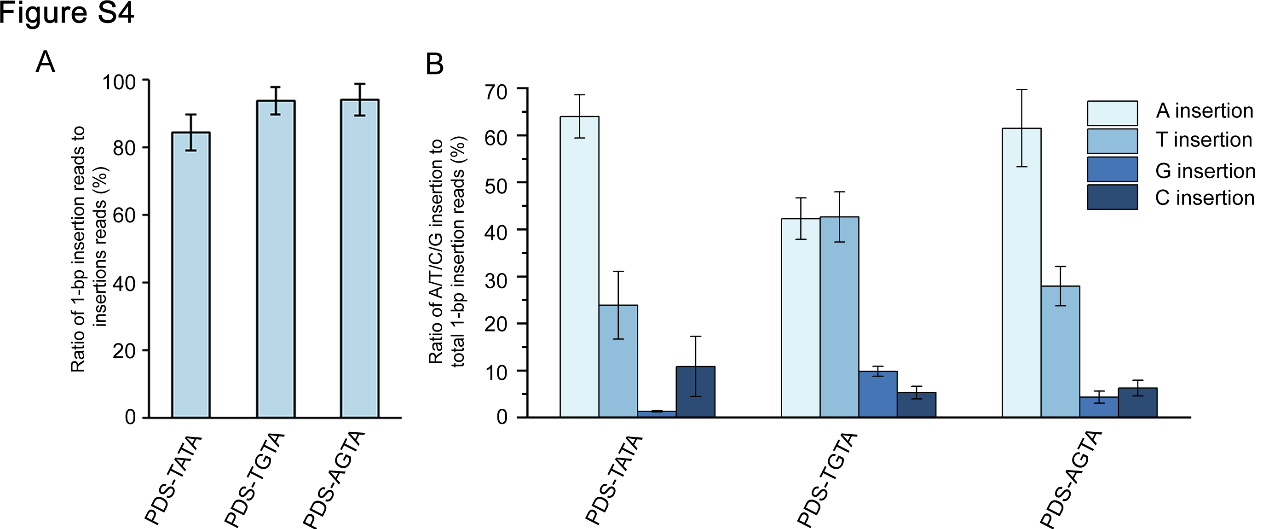
**

**Supplemental Figure S4.** Outcome preference of FrCas9-mediated editing in rice calli.

The preferences of the 1-bp insertion (A) and inserted nucleotide (B) were analyzed for outcomes at the PDS-TATA, PDS-TGTA, and PDS-AGTA sites. The error bars indicate the standard deviations of three independent replicates.

**Supplementary Table S1.** Determining potential off-target mutations mediated by FrCas9 in transgenic plants.

| sgRNA | Off-target Site^#^ | Mismatched bases (bp) | Genome location | No. of plants | Off-target  Mutants |
| --- | --- | --- | --- | --- | --- |
| PDS-T | OFF-1 | 5 | Chr08: 23936643 | 48 | 0 |
|  | OFF-2 | 4 | Chr08: 26034563 | 48 | 0 |
|  | OFF-3 | 5 | Chr08: 967184 | 48 | 0 |
| PDS-AGTA | OFF-1 | 5 | Chr08: 10270239 | 48 | 0 |
|  | OFF-2 | 5 | Chr08: 13374438 | 48 | 0 |
|  | OFF-3 | 5 | Chr08: 18217915 | 48 | 0 |
|  | OFF-1 | 5 | Chr05: 28452899 | 48 | 0 |
| PDS-TGTA | OFF-2 | 5 | Chr01: 17168446 | 48 | 0 |
|  | OFF-3 | 3 | Chr02: 860430 | 48 | 0 |
|  | OFF-1 | 5 | Chr05: 28933189 | 48 | 0 |
| PDS-CCTA | OFF-2 | 5 | Chr01: 12320455 | 48 | 0 |
|  | OFF-3 | 5 | Chr01:22049375 | 48 | 0 |

^#^, three most likely off-target sites were predicted by Cas-OFFinder for screen.

**Supplemental Table S2.** FrCas9-mediated bidirectional editing with a single PAM in transgenic rice plants.

| targets | Examined plants | WT | Single mutants | Double mutants |
| --- | --- | --- | --- | --- |
| HD1-T1 | 24 | 6 | 6 | 10 |
| HD1-T2 |  |  | 2 |  |

**Supplemental Table S3.** Editing conducted by FrCas9-based BEs in T_0_ plants.

| Base editors | Targets | PAM | Examined plants* | Mutants^#^ | | | |
| --- | --- | --- | --- | --- | --- | --- | --- |
|  |  |  |  | Total (%) | Ho/Bi | He | Ch |
| A3A-FrBE3 | NAL1-T | AGTA | 48 | 47.9 | 2 | 15 | 6 |
|  | HD1-T3 | AGTA | 48 | 4.2 | 0 | 2 | 0 |
| FrABE8e | HD1-T4 | AGTA | 48 | 8.3 | 0 | 4 | 0 |
|  | BADH2-T | TGTA | 48 | 12.5 | 0 | 4 | 2 |

*, For each vector, 48 independent T_0_ lines were randomly selected from regenerated rice plants.

#, Ho, homozygous, only a mutation was detected in the line; Bi, bi-allelic, the plant carried two types of mutation; He, heterozygous, the plant simultaneously carried a mutation allele and a wild type allele at the target site; Ch, chimeric, the plant harboring more than two genotypes at the target site.

**Supplemental Table S4.** Oligos and primers used in this study.

1. Sequence of rice genomic sites for genome editing and base editing.

| **Target** | **gRNA Sequence (5’ to 3’)** |
| --- | --- |
| PDS-T | TCTGAGTGGATGAAAAAGCAGG |
| SWEET13-T | CCCACCTAGCTAGCTTTGCTCC |
| PDS-AATA | TTAGCTGGTTTATCAACGGCAA |
| PDS-ATTA | ACAGTGAACAACCCACTAAACC |
| PDS-ACTA | TGACACTGAAATCATCGAAGCA |
| PDS-AGTA | TCCTACTCATAGGTGCTTCGCA |
| PDS-TATA | GTGATTGCTGGAGCAGGTATGA |
| PDS-TTTA | ATCATATGCAGCGCTGGAGTTG |
| PDS-TCTA | GATTCCGTCGGAGGCTCGGTGC |
| PDS-TGTA | GGTGCTCTACAGGTTCAACCTT |
| PDS-GATA | TCGTGATTGCTGGAGCAGGTAT |
| PDS-GTTA | TGGATGGTAATCCTCCTGAAAG |
| PDS-GCTA | CCATACTAAGAAACAATGAAAT |
| PDS-GGTA | ACAGGTCGTGATTGCTGGAGCA |
| PDS-CATA | TTCATTGTTTCTTAGTATGGCC |
| PDS-CTTA | CCAGCAATGGTTGGTGGCCAAG |
| PDS-CCTA | TCCTCCTGAAAGGTTATGCATG |
| PDS-CGTA | TGGTGAGGTTCGGCTGAATTCT |
| BADH2-TGTA/BADH2-T | AGGTAATAATCTACTACACGGT |
| ALS-TGTA | TATGTATGCGCCCTATTCGCCT |
| SLR1-AGTA | CCCAGCCGCTGCCGACTCGTCG |
| NAL1-AGTA | AATCCTTTCTGAAGGTTTCCAA |
| CDC48-CCTA | GGAGATATTCTGTGATGGTGAG |
| DOFT-CCTA | AACGGCGGGTTCGAGGAGGGCC |
| WX-T | GGTGGTGGTGTGGGTGGCTATT |
| HD1-T1 | GCCTCTACGGAACTGCTGTATA |
| HD1-T2 | TTTATTCCATTTGATGAGTGCG |
| NAL1-T | GAATTCCTCAACGCGGGGATGT |
| HD1-T3 | CACTGCCTCTACGGAACTGCTG |
| HD1-T4 | GTAGTAACAACTAACTAACAAC |

1. Primers for Hi-TOM analysis on the targets

| **Target** | **Sequence** |
| --- | --- |
| PDS-T | FP: ggagtgagtacggtgtgcGGTGGCCAAGCTTATGTTGA |
|  | RP: gagttggatgctggatggCTTCTCCTATATCCATTCCA |
| PDS-TGTA | FP: ggagtgagtacggtgtgcCAGCATGTGAGCTTTGGAGTGA |
|  | RP: gagttggatgctggatggCAACCTGCAGAAAAGCCAATCA |
| PDS-AGTA | FP: ggagtgagtacggtgtgcGCTGCCTGTCATCTATGAACAT |
|  | RP: gagttggatgctggatggGGTTGAACCTGTAGAGCACCGA |
| PDS-CCTA | FP: ggagtgagtacggtgtgcATGCAAATGTGTAGGAGAAGCA |
|  | RP: gagttggatgctggatggAATACGAGAATTCAGCCGAACC |
| SWEET13-T | FP: ggagtgagtacggtgtgcTCTATGATTACTTTGATGCG |
|  | RP: gagttggatgctggatggAGCTCTGAAGAAGAGTTCAA |
| BADH2-TGTA/BADH2-T | FP: ggagtgagtacggtgtgcTGTATCTACCGCCAAAAGCC |
|  | RP: gagttggatgctggatggAACATACAAGATTATTTCTA |
| ALS-TGTA | FP: ggagtgagtacggtgtgcATTGATCCGCATTGAGAACC |
|  | RP: gagttggatgctggatggTGAACCCCTTAGCAATAGTC |
| SLR1-AGTA | FP: ggagtgagtacggtgtgcCGCCATGCTTCCACCTCGTC |
|  | RP: gagttggatgctggatggCCGACGGGTCAGCCGTCGC |
| NAL1-AGTA | FP: ggagtgagtacggtgtgcTTCTTATGGCCGACATCCAA |
|  | RP: gagttggatgctggatggTCATCAAGTCAAGCAAGCTA |
| CDC48-CCTA | FP: ggagtgagtacggtgtgcGGGTGGGATGAGAAGTGTTG |
|  | RP: gagttggatgctggatggATCATCGTAACCAACTTCAT |
| DOFT-CCTA | FP: ggagtgagtacggtgtgcCGTCGCCATCAGGGCGCTGT |
|  | RP: gagttggatgctggatggGTCCTCGATGTTGGGCGGGA |
| WX-T | FP: ggagtgagtacggtgtgcGACCGGGTAAAATGTGTTGC |
|  | RP: gagttggatgctggatggTATTTGTGTCTTGAGATAGA |
| HD1-T1/T2/T3 | FP: ggagtgagtacggtgtgcCCACTCTTCAGTCACATAGG |
|  | RP: gagttggatgctggatggTTACCCACAAGTGGTTCTTT |
| NAL1-T | FP: ggagtgagtacggtgtgcCTCCGTCACCTTCACCGCCA |
|  | RP: gagttggatgctggatggGAGTCCGTCCTCGACCAACA |
| HD1-T4 | FP: ggagtgagtacggtgtgcAGCAACAGCGTGAGTTCATC |
|  | RP: gagttggatgctggatggCCACTCAAAAGTACTAGTAT |

1. Primers for Sanger sequencing analysis on the targets

| **Target** | **Sequence** |
| --- | --- |
| PDS-T | FP: GATCATGCGTGCATTTAACA |
|  | RP: AACTGCCAGTTCTTCCCAAT |
| PDS-TGTA | FP: CTCATAGGTGCTTCGCAAGT |
|  | RP: TCATCCTTCCAAGCAGCTAT |
| PDS-CCTA | FP: TTTCCGGAGTAGTACCTTAC |
|  | RP: AGTTCATAAACCAGTCATAG |
| SLR1-AGTA | FP: CGTCGCGCAGAAGCTGGAGC |
|  | RP: AACCACCACAACCGGCACGG |
| WX-T | FP: TACGCGAGTGCATGCAGATG |
|  | RP: TAAACATATATGTATAACTT |
| HD1-T1/T2/T3 | FP: CCACGTGTCCATCGAACTGT |
|  | RP: GAAGTGGTGCTATTAGAGCA |
| NAL1-T | FP: CCAGCAACGACAAGGCTGAT |
|  | RP: GAAGTGGAGGTGCCCGAAGC |
| HD1-T4 | FP: GTGTGGTACCTTCACAGATC |
|  | RP: CAGGAAGTAGTCAACTGGTC |
| BADH2-T | FP: GACCCCATGGCACTTTAATG |
|  | RP: CCTCAGTTAATCTCTGGCAT |

4. Primers for amplicon deep sequencing

| **Target** | **Sequence** |
| --- | --- |
| PDS-ATTA/GGTA/GATA/TATA | FP: TGCCAGGACTTTCCAAGACC |
|  | RP: ACCACGATGTGACTGCTATCA |
| PDS-AGTA | FP: ATGGATACTGGCTGCCTGTC |
|  | RP: ACCGAGCCTCCGACGGAATC |
| PDS-AATA | FP: TAACTTTGATAGCAGTCACA |
|  | RP: TTAAATGCAACATAAAAGCA |
| PDS-ACTA | FP: TAAAGACATGGATGCCTACT |
|  | RP: CCTCACCTTGGTGTCTTCAC |
| PDS-TCTA/TGTA | FP: AACTGGAACCAGCCAAGCAAG |
|  | RP: CAAACAACCTGCAGAAAAGCC |
| PDS-GCTA/CATA/CTTA | FP: GCCATGGAGCTTTTATGTTGTCA |
|  | RP: ACCTGCTTTTTCATCCACTCAG |
| PDS-GTTA/CGTA/CCTA | FP: TTATGCAAATGTGTAGGAGA |
|  | RP: TCACCTGGTGTTGCAAAAACA |
| PDS-TTTA | FP: GTTTGGCGAGCTTGGTATTA |
|  | RP: CCACCAATACGATGTACTTCTG |

5. Primers for Hi-TOM analysis of off-target analysis.

| **Target** | **Sequence** |
| --- | --- |
| PDS-T | OFF1-FP: ggagtgagtacggtgtgcTTCACCCATCCTACACATAGCAA  OFF1-RP: gagttggatgctggatggTTAGGAATCATTGTATCGAAGTTTGTAA  OFF2-FP: ggagtgagtacggtgtgcGGTTGGACATGAGCCTTGTAGTT  OFF2-RP: gagttggatgctggatggGCGCAGAGAAAACAAGAAATTTTCTC  OFF3-FP: ggagtgagtacggtgtgcTCTCTCGTGCAAATACTTTGTAGA  OFF3-RP: gagttggatgctggatggTACCCTCCTTTATCAAATTCCAATGTA |
| PDS-AGTA | OFF1-FP: ggagtgagtacggtgtgcATTGGGACAGTACGGTAGAAAT  OFF1-RP: gagttggatgctggatggGCTAGATAACTTAATCAGCTAGGT  OFF2-FP: ggagtgagtacggtgtgcAGAACCACTATACAGAGGTACCAC  OFF2-RP: gagttggatgctggatggGGGTGGCTAGTACTGTTCCAA  OFF3-FP: ggagtgagtacggtgtgcCGCCCGTTCTTGGTACTTCAACTTA  OFF3-RP: gagttggatgctggatggTTCGCCCAGCCTTTGGCGAAT |
| PDS-TGTA | OFF1-FP: ggagtgagtacggtgtgcTTATCAAATACCTTCACCGCTTCG  OFF1-RP: gagttggatgctggatggATGTCCGGAGCTATGATATGGG  OFF2-FP: ggagtgagtacggtgtgcAGATGAAGAAACCTATGCTTTGCT  OFF2-RP: gagttggatgctggatggCCTTTCCATTTGTGCGCTTAG  OFF3-FP: ggagtgagtacggtgtgcGGCATGGCTATGGTGCAGTAG  OFF3-RP: gagttggatgctggatggCACACCCCAGGTGGTAGGTT |
| PDS-CCTA | OFF1-FP: ggagtgagtacggtgtgcCATCAAGCTATCGTCAGCGGTG  OFF1-RP: gagttggatgctggatggGTAGTCGTGTAGATATTGCGGCTG  OFF2-FP: ggagtgagtacggtgtgcGCACTATAAGGACTACTCGGAT  OFF2-RP: gagttggatgctggatggGCTTATCTTGGATTGTCTAGCTCT  OFF3-FP: ggagtgagtacggtgtgcCTGTTCAATATATATGGCAGGCA  OFF3-RP: gagttggatgctggatggTTAGAACTTATCAAATGGTACCATGTT |

**Supplemental Sequence**

> rice codon-optimized FrCas9

ATGGCCCCAAAGAAGAAGCGCAAGGTCTGCACAAAGGAGTCTGAGAAACTCAACAAGAATGCCGACTACTACATTGGCCTTGACATGGGGACGTCCTCCGCCGGCTGGGCTGTTTCAGATAGCGAATACAATCTTATACGGCGGAAGGGAAAAGATCTGTGGGGAGTCAGGCAGTTTGAAGAGGCGAAAACGGCCGCAGAGCGCAGAGGATTCCGCGTCGCTCGTCGCCGGAAGCAACGTCAGCAGGTCAGAAACCGGCTTCTATCGGAGGAGTTCCAGAACGAGATCACCAAGATTGATTCGGGATTTCTCAAGCGCATGGAGGACTCGCGTTTCGTCATCAGCGACAAGAGGGTTCCTGAGAAGTACACGCTGTTTAACGACTCGGGTTATACTGATGTGGAATATTACAACCAGTACCCGACGATATACCATCTTAGGAAGGCGCTGATTGAGAGCAACGAAAGATTTGATATTAGGCTCGTGTTCTTGGGCATTCACTCTCTATTTCAGCATCCGGGGCATTTTCTGGACAAAGGAGATGTTGACACCGACAACACGGGTCCGGAGGAACTCATCCAGTTCCTAGAGGACTGCATGAATGAGATCCAAATTTCCATCCCACTCGTGTCCAATCAAAAAGTACTGACTGACATTCTTACAGACAGTAGGATCACACGACGCGACAAGGAGCAGCAAATACTCGAAATCCTACAACCTAACAAGGAAAGTAAGAAGGCTGTATCGCAATTTGTTAAGGTATTGACTGGACAGAAAGCTAAGCTAGGCGACCTGATCATGATGGAAGATAAGGACACCGAGGAGTACAAGTATTCTTTCTCCTTTAGGGAGAAGACTCTCGAAGAGATACTACCGGACATCGAGGGCGTCATAGACGGGTTGGCGTTGGAGTATATTGAAAGTATCTACTCCCTCTACTCGTGGTCCCTCTTAAACTCATACATGAAGGATACTCTGACGGGCCACTACTACTCTTATTTAGCTGAGGCGCGAGTGGCGGCGTACGACAAGCATCACTCCGATCTTGTAAAGTTGAAAACTCTCTTCAGGGAATATATCCCTGAAGAGTACGACAATTTCTTCCGTAAGATGGAAAAGGCTAATTACAGCCATTACATCGGTTCCACGGAGTATGATGGAGAAAAGAGATGTAGGACGGCCAAAGCGAAGCAAGAGGATTTCTATAAGAGTATAAACAAAATGCTCGAAAAGATCCCCGAGTGTAGTGAGAAAACAGAAATACAGAAAGAGATCATTGAGGGGACCTTTTTGTTGAAGCAAACCGGACCGCAGAATGGCTTTGTGCCCAACCAGCTCCAGCTCAAAGAGCTGCGGAAGATATTGCAGAATGCATCAAAGCACTATCCTTTTTTGACAGAGAAGGACGAACGGGATATGACAGCAATTGACCGCATTGAGGCATTATTCTCCTTTAGGATCCCGTATTACATCGGCCCACTCAAGAATACAGATAACCAGGGTCATGGCTGGGCGGTGAGGCGCGATGGCCATGAACAGATTCCGGTGAGGCCGTGGAACTTCGAGGAGATTATTGATGAGAGTGCATCTGCGGATCTATTCATCAAAAACCTGGTTAACAGCTGCACGTATCTTCGTACCGAGAAAGTTCTCCCCAAGAGCAGTCTCCTGTACCAGGAGTTTGAGGTTCTTAATGAACTGAATAATCTGCGCATAAACGGGATGTACCCAGATGAGATTCAGCCAGGTCTTAAGAGGATGATTTTTGAGCAGTGTTTCTACTCAGGAAAAAAGGTCACCGGCAAGAAGCTCCAGCTCTTTCTCAGGTCAGTCTTGACGAACAGCTCGACGGAAGAATTTGTACTTACAGGTATCGATAAAGATTTCAAGTCCTCCCTCTCCTCCTACAAGAAGTTCTGCGAGTTGTTCGGCGTGAAGACTCTCAATGACACACAAAAGGTGATGGCGGAGCAAATCATAGAATGGTCAACCGTCTATGGCGACAGCAGGAAATTTCTGAAAAGAAAGCTGGAGGATAACTATCCAGAGTTGACAGATCAACAGATCCGAAGGATCGCTGGCTTCAAGTTTTCCGAGTGGGGGAACCTCTCCCGGGCATTCCTCGAAATGGAGGGTTATAAGGATGAAGCCGGGAATCCCGTGACAATAATTCGCGCCCTGCGTGACACGCAGAAGAATCTCATGCAGCTGCTCTCGAACGATTCAGCATTTGCCAAGAAACTTCAAGAATTGAATGATTACGTGACCAGAGATATTTGGAGCATCGAGCCCGACGACCTGGACGGCATGTATCTTTCAGCCCCTGTTCGTCGGATGATCTGGCAGACTTTCTTGATACTGCGCGAGGTTGTTGACACTATCGGCTACTCGCCGAAGAAGATTTTCATGGAGATGGCCAGGGGCGAACAGGAAAAAAAGAGAACGGCGAGCCGTAAGAAGCAGTTGATTGACCTCTACAAGGAAGCAGGAATGAAAAATGATGAACTTTTCGGTGATTTAGAATCACTTGAAGAAGCACAACTTAGGTCTAAGAAGCTGTATCTCTACTTCAGACAGATGGGGAGGGATATATACAGTGGGAAGTTGATTGATTTTATGGATGTATTGCACGGCAACAGATATGATATCGACCACATCCATCCACAGAGTAAAAAGAAAGATGATTCTCTCGAAAACAATCTCGTTCTGACCAGTAAGGATTTCAACAACCATATCAAGCAAGACGTCTACCCCATACCTGAACAAATACAATCCCGCCAAAAAGGATTCTGGGCTATGCTTCTTAAGCAAGGCTTCATGTCCCAGGAGAAGTACAATCGCCTCATGAGGACAACACCTTTCACCGACGAGGAACTCGCGGAGTTCGTGAATCGACAGCTCGTGGAGACTAGACAAGGAACTAAAGCTATTATCTCTCTAATAAATCAATGCTTCCCTGATAGCGAGGTGGTGTATGTTAAAGCCGGGAATACCAGCGACTTCCGACAGCGGTTCGACATACCGAAGAGCAGGGATTTGAATAACTATCACCACGCCGTCGACGCCTACCTGAACATCGTCGTGGGCAATGTCTACGACACGAAGTTCACAAAGAACCCTATCAATTTTATCAAGAAGATGCGTAAGTCCGGTAACCTCCACAGCTATTCCCTGCGCCGGATGTATGACTTCAATGTTCAGCGCGGTGACCAAACCGCCTGGGTGGCTGAAAATGACACCACCCTCAAGACGGTGAAGAAGACCGCATTTAAGACATCTCCCATGGTAACAAAGAGGACCTACGAGAGAAAGGGGGGGCTGGCAGATTCGGTCCTCATCGCCGCCAAAAAAGCAAAGCCCGGTGTGCACCTCCCGGTCAAAACCTCTGATTCACGCTTCGCCAACCAGGTGTCAACATATGGCGGATATGATAATGTGAAGGGTTCCCACTTCTTTCTTGTTGAGCATCAGCAAAAGAAAAAGACAATTCGCTCGATTGAGAATGTCCCAATACATCTAAAAGAAAAGCTCAAGACCAAAGAAGAGCTGGAGCACTACTGCGCGCAGGTTTTAGGTATGGTTCAGCCGGACGTTAGACTCACTCGCATCCCAATGTATAGTCTTCTCCTTATCGATGGCTACTATTATTACCTGACTGGCCGGACTGGCGGCAATTTGAGCCTGTCAAATGCCGTGGAGCTGTGTCTCCCTGCCAAAGAACAAGCGCACATTCGGATGATCAGTAAAATTGCCGGTGGACGGTCTACTGATGCTCTCTCAGCTGAGGCTAAAGACGACTTTCGAAAAAAGAACCTAAGGCTGTACGATGAGCTGGCCGAGAAGCATAGATCCACGATCTTCAGCAAACGGAAAAACCCAATAGGACCAAAACTGTTAAAATACAGAGAGGCGTTCGTGAAGCAAACAATTGAGAATCAGTGTAAGGTGATACTCCAGATCCTTAAATTGACCTCTACAAACTGCAAAACTTCCGCCGACCTGAAGTTAATTGGGGGGTCTGGGCAAGAAGGAGTGATGTCTATCTCAAAGCTACTCCGCGCCGAGAAATATGCCGAGTTCTACTTAATATGCCAGTCCCCGTCGGGTATTTATGAGACACGCAAGAACTTATTAACTATCTCCGGCGGCAGCCCAAAGAAGAAGCGGAAGGTGTCTGGAGGTTCTCCTAAGAAAAAGAGAAAAGTGTCCGGCGGCTCCCCGAAGAAGAAGCGCAAGGTGTGA

Blue, SV40 NLS;

Black, FrCas9
